# Air pollution in New Delhi is more severe than observed due to hygroscopicity-induced bias in aerosol sampling

**DOI:** 10.1038/s44407-024-00001-6

**Published:** 2025-03-12

**Authors:** Ying Chen

**Affiliations:** School of Geography, Earth and Environmental Sciences, University of Birmingham, Edgbaston, Birmingham, B15 2TT UK

**Keywords:** Environmental sciences, Environmental chemistry, Environmental impact

## Abstract

New Delhi, India, is suffering from one of the worst air quality in the world, estimated to be responsible for 10,000 premature deaths per year. Although the high pollution level of fine particulate matter (PM_1_) in New Delhi has attracted global attention, the true level of PM_1_ pollution could still be underestimated due to the inherent sampling bias associated with particle hygroscopic growth. This study compiles a comprehensive in-situ observation dataset from a series of recent studies in New Delhi, to quantify hygroscopicity-induced bias for the first time, and found that the more severe pollution the larger underestimation, and report the underestimate can be up to 20% (or 50 µg/m^3^) of PM_1_ concentration on average in humid winter morning rush hours. This study fills in the gap of the understanding of PM_1_ pollution in the most polluted megacity in the world, and provides a calibration approach for future studies to develop better understanding of air quality in New Delhi.

## Introduction

New Delhi, the capital of India, has been recognised as the most polluted capital city all over the world^1^. As the second largest city in the world and its fast growth of economy and urbanization, this severe air quality problem is threatening the public health of ~33 million citizens in New Delhi. Fine particulate matter is the most dominant pollutant in New Delhi^2–4^, and is estimated to be responsible for about 10,000 premature deaths per annual^3,5^. The up-to-date World Health Organization (WHO) Ambient Air quality database reported the annual average of fine particulate matter mass concentration of 121 µg/m^3^ in New Delhi, which is 24 times higher than the suggested healthy level by the WHO air quality guideline^1^.

This severe air pollution problem in New Delhi has captured wide attentions from the scientists all over the world. Vast of short-term comprehensive/intensive observations^6–8^ and long-term regular monitoring networks, for example Central Pollution Control Board (CPCB)^9^ and SAFAR^10,11^ networks, have been developed in recent year to better understand the air pollution and to suggest for mitigation strategies^12,13^. Using the CPCB network observations, ref. ^5^ reported the record-high hourly concentration of fine particulate matter in New Delhi of up to 1676 µg/m^3^ during Diwali Festival under an unfavourite stable meteorological condition, this is 70% higher compare to the worst record in Beijing, China^14^.

**Fig. 1:**
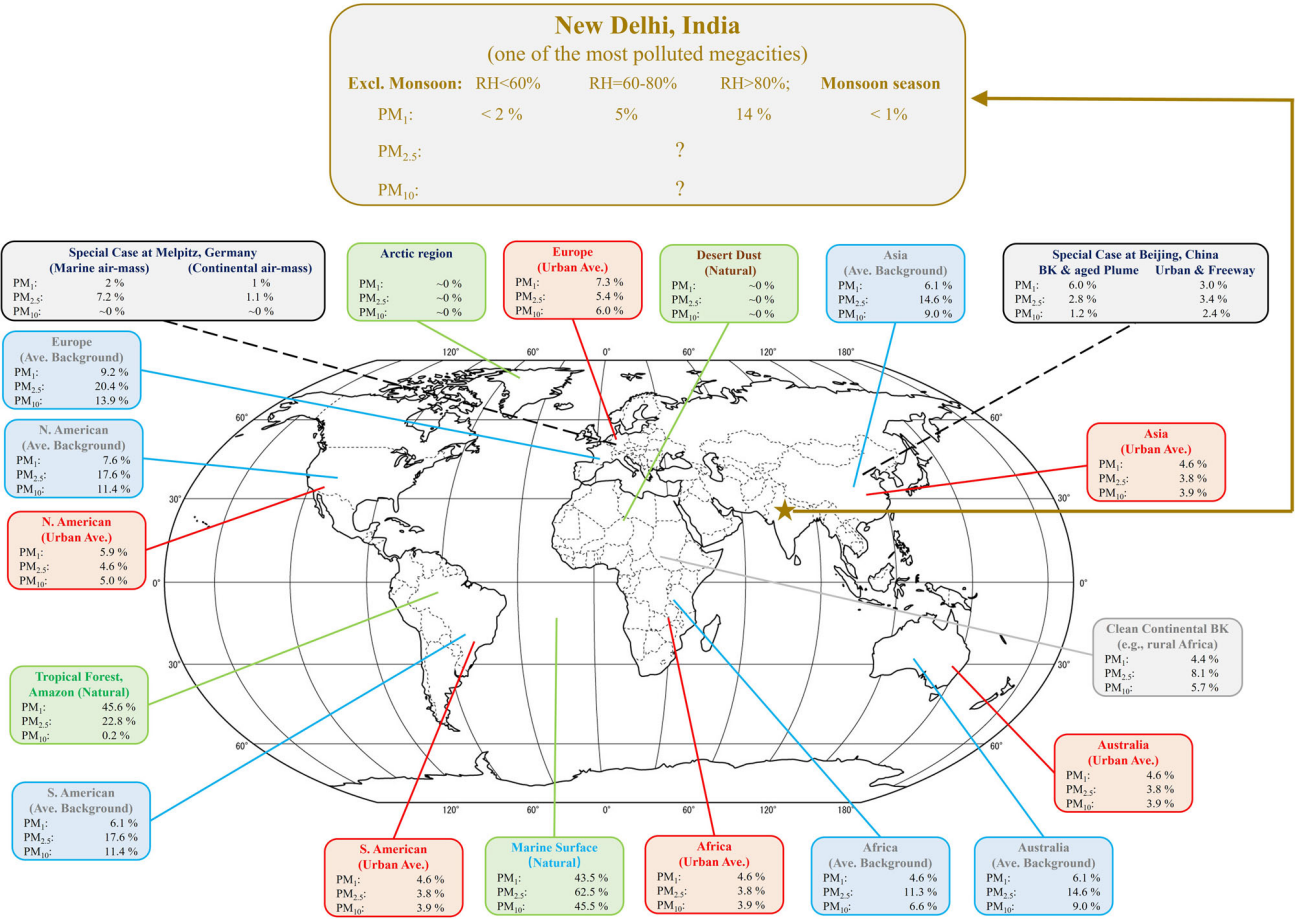
Global summary of sampling cut-off shift induced underestimation of particulate matter pollution. This study contributes the estimate for New Delhi, India (dark yellow). This figure is modified from ref. ^15^, which provides the data of other places (re-use under CC-BY 4.0 License).

The reported particulate matter concentration in New Delhi is breath-holding, however, we may still underestimate it. This is because hygroscopic growth of particulate matter can effectively reduce its sampling efficiency and therefore lead to underestimate of its mass concentration. Hygroscopic growth leads to particle size swelling, resulting in a cut-off shift effect therefore reduces the collection efficiency of sampling inlets^15^. This bias is inherent in particle sampling and commonly exist all over the world; and this bias is largely dependent on the water uptake ability of aerosol particles: the higher aerosol hygroscopicity the larger underestimation in mass concentration. For example, the concentration of particulate matter could be underestimated by more than 40% over marine regions where have plenty of highly hygroscopic sea-salt particles; while, it was believed to be lesser than 5% in Asian cities (see the Fig. 1) where particles are believed to be much less hygroscopic. However, recent observational studies have reported for the first time that particulate matter in New Delhi is extremely hygroscopic^16–18^, with associated daily water content reach up to 740 µg/m^3^ ^16^, indicating large underestimation of particulate matter concentration in New Delhi.

This hygroscopic growth of particles, the associated extra cut-off during sampling and hence the underestimation of dry PM_1_ concentration have been well recognised; the estimate of its impacts have been reported over Europe, America, Africa, and China^15^. However, this important aspect for precise understanding of air pollution is still untouched over India, the country with the biggest population suffering hazardous air pollution, due to lack of observations. The recent in-situ long-term observations of detailed physicochemical properties of particulate matter in New Delhi^6,7,19^ enable quantification of this sampling bias in particulate matter observations for the first time. Here I compile the comprehensive observations from a supersite in New Delhi and adopt the state-of-the-art thermodynamic theories to provide a more unbiased figure of particulate matter pollution in New Delhi.

## Results and discussion

### Diurnal and seasonal variation of underestimation in PM1

Table 1 shows the diurnal and seasonal variation of the sampling underestimation of PM_1_ mass concentration in New Delhi. One can observe that the underestimation can reach up to ~20% in winter (December to January) morning rush hour (8–9am). This period is usually the most polluted one throughout the year, due to a combination of shallow planet boundary layer, abundant human activities and emissions especially from traffic, and a humid condition favouring secondary formation^5^. The morning rush hour is also the most humid time window in the diurnal evolution in all seasons, possibly because surface water starts to evaporate after sun rise and human activities (e.g., breakfast cooking) and the evolution of planet boundary layer is slightly lagged lead to accumulation of water vapour in the near surface layer. This humid condition also enhances hygroscopic growth of particles and therefore lead to large sampling underestimation. Our analysis shows that the average RH in winter morning rush hour is 90% with the highest growth factor during the whole year of 1.55. The average PM_1_ during winter morning rush hour is ~250 µg/m^3^, after the correction of sampling bias it can reach up to 300 ug/m^3^. Similarly, the spring (February to March) morning rush hour with RH of 80% on average, leading to the second highest growth factor of 1.43 and correspondingly 8.6% underestimate due to cut-off shift. The average PM_1_ during spring morning rush hour is ~210 µg/m^3^, after the correction of sampling bias it can reach up to 230 µg/m^3^. This indicates that the particulate matter pollution in New Delhi is much more severe than we thought.

**Table 1:** ALWC-induced particle swelling and the corresponding extra cut-off by sampling inlets, with extra cut-off >5% shown in bold

	Characteristic Times	Particle Number Size Distribution^*^		RH [%]	PM_1_ [µg/m^3^]	Cl^-^ [µg/m^3^]^*^	Growth Factor	cut-off diameter [nm]	cut-off mass^#^ [%]
		D_gm_ [nm]	σ_g_						
Spring	Average	106.0	1.90	53.7	132.7	12.8	1.16	859	4.4
	02:00–03:00	122	1.8	65.0	167.8	17.2	1.19	839	3.3
	**08:00**–**09:00**	122	1.8	79.4	212.2	31.1	1.43	698	**8.6**
	14:00–15:00	100	2	32.3	62.3	1.6	1.05	957	1.4
	20:00-21:00	80	2	44.6	112.1	4.7	1.06	943	1.8
Summer	Average	100.0	1.83	38.5	64.2	1.5	1.09	913	0.9
	02:00-03:00	100	1.8	43.8	72.9	1.4	1.11	899	1.2
	08:00-09:00	100	1.9	50.1	78.8	4.4	1.14	874	2.7
	14:00-15:00	111	1.7	28.6	45.8	0.5	1.07	935	0.3
	20:00-21:00	89	1.9	32.8	66.7	0.5	1.07	936	1.2
Monsoon	Average	94.2	1.68	75.0	90.0	0.4	1.19	842	0.3
	02:00–03:00	101	1.8	82.4	115.4	0.6	1.18	845	1.8
	08:00–09:00	110	1.8	85.2	102.4	0.7	1.25	803	3.2
	14:00–15:00	90	1.4	64.0	75.2	0.2	1.14	876	0.00
	20:00–21:00	76	1.7	71.1	74.4	0.4	1.14	878	0.1
Winter	**Average**	114.2	1.85	70.9	223.0	23.1	1.29	777	**6.6**
	**02:00**–**03:00**	122	1.8	82.9	269.1	33.8	1.42	705	**8.5**
	**08:00**–**09:00**	122	1.9	90.1	251.2	38	1.55	644	**19.2**
	14:00-15:00	125	1.8	47.9	136.3	6.1	1.13	886	2.5
	20:00-21:00	88	1.9	65.5	245.7	19.7	1.19	837	2.7

^*^ Geometric mean diameter (D_gm_), standard deviation (σ_g_) of fine particle number size distribution (PNSD), and chloride are sourced from Gani et al.^6,24^

^#^ sampled fine particle mass loss due to cut-off shift of more than 5% is shown in bold.

In monsoon season (July to September), despite of humid condition (e.g., RH = 85% in morning rush hour), moderate growth factors (1.14–1.25) are found. This is because that uniquely high chloride concentration is the main reason of high aerosol hygroscopicity in New Delhi^16,17^; however, chloride (such as hydrogen chloride and ammonium chloride) is highly soluble and washed out by the heavy and frequent precipitation in monsoon season, leading to very limited ( < 1%) chloride mass fraction and therefore the lowest aerosol hygroscopicity within the year (see Table 1 and supplementary Fig. 1)^16^. In addition, wet deposition is the main sink of accumulation mode particles (100–1000 nm), the precipitation also wash out larger particles in PM_1_, leading to the smallest geometric mean diameter of only 94 nm averaged over monsoon season. The smaller size of particles and moderate hygroscopicity during monsoon lead to nearly negligible influence ( < 5%) of cut-off shift on particles sampling.

Summer (April to June, or pre-monsoon) is the driest season during the year, with 28% < RH < 50%. This largely inhibits the hygroscopic growth of particles, with growth factors of 1.07–1.14; therefore, negligible influence ( < 3%) of cut-off shift on particles sampling is observed. The strong solar and hot temperature in summer also lead to high planet boundary layer, facilitating dilution of air pollution and therefore the cleanest season with average PM_1_ of 64 µg/m^3^.

### Large underestimation in humid & severe polluted conditions

**Fig. 2:**
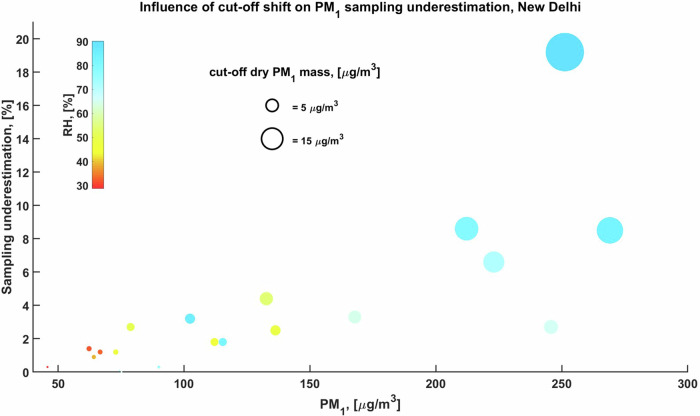
Influence of particle hygroscopic growth induced cut-off shift on PM1 sampling underestimation, in New Delhi. Y-axis indicates the extra cut-off percentage of mass in dry PM1. Size of the dots indicates the mass of dry PM1, which is cut-off during sampling due to hygroscopic growth and therefore underestimated.

**Fig. 3:**
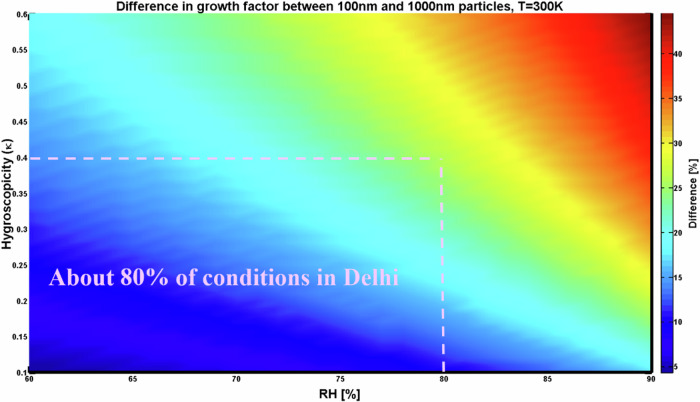
Difference in growth factors between 100 nm and 1000 nm particles. The information of aerosol hygroscopicity in Delhi is sourced from refs. ^18,19^, and the calculation follows κ-Köhler theory^27^.

As one can see from Fig. 2 that the underestimation of PM_1_ due to sampling cut-off shift is more significant when pollution level is higher. Dry PM_1_ mass concentration is generally underestimated by only less than 5% when PM_1_ < 150 µg/m^3^, but it can reach up to 10–20% (or 10–50 µg/m^3^) when PM_1_ > 200 µg/m^3^. High humid not only exacerbates the particulate matter pollution in New Delhi^16,17^, but also results in more remarkable underestimate of pollution levels. For example, when RH increase from 65% to 90%, the underestimation of PM_1_ pollution can be enlarged exponentially from ~2% (5 µg/m^3^) to ~20% (50 µg/m^3^), see Figs. 2–3. This suggests that the severe pollution episodes could be more rigorous than we thought, extra attention should be paid to precautions of the warning of severe pollution in New Delhi to better serve public health, especially in humid days; it is note worth that humid condition frequently occurs in New Delhi with frequency of RH > 80% of ~23% throughout the year and of ~44% in winter^16^.

## Discussion

This study finds particulate matter pollution in New Delhi could be remarkably underestimated due to particle hygroscopic growth, despite of global attention attracted by its severe pollution. This study provides the first estimate of this bias and characterises it for different pollution levels, environments, diurnal and seasonal patterns. I provide a summary table (Table 1) for correction of this bias and hence facilitate more precise and better understanding of air pollution in New Delhi. In general, i) the level of this bias ( < 5%) in New Delhi is in line with other urban area all over the world when RH < 80%; ii) monsoon season is negligibly influence by this bias ( < 1%) because of fast wash out of hygroscopic/soluble species; iii) significant underestimate of PM_1_ by about 15% on average is found when RH > 80% (about one quarter of time throughout the year), see details in Fig. 1. Anthropogenic chloride largely enhances the particle growth under humid conditions in New Delhi^16,17^, contributing 40–50% of aerosol liquid water (ALWC) in spring and winter (the most polluted two seasons, supplementary Figs. 1-2); this could be the reason of the uniquely high bias in humid conditions compared to other cities worldwide. Open biomass burning and residential emissions are the major sources of chloride in India^20^, suggesting that control of these emission sources would not only improve air quality but also help reduce bias in particle observations and hence facilitate better understanding of air pollution. A limitation of this study is that I only estimate the bias in PM_1_ observations, while, the biases in PM_2.5_ and PM_10_ is not analysed (Fig. 1), due to the lack of data. Nevertheless, I expect the influence of this bias on PM_2.5_ and PM_10_ is much smaller than on PM_1_. This is because the majority of particulate chloride is exist in submicron particles which provide the largest surface area for its secondary formation; and because super-micro particles, with many sources from resuspended dust, desert dust and tyre/break wear in New Delhi^21,22^, are relatively hydrophobic. We call for more in-situ studies of detailed physicochemical properties for PM_2.5_ and PM_10_, in order to have a more holistic understanding of air pollution and advise for mitigation in New Delhi.

## Methods

### Data and materials

Comprehensive in-situ observations of fine particulate matter, with diameter < 1 µm (hereafter PM_1_), have been performed in a supersite in New Delhi during January 2017 to March 2018. The detailed PM_1_ chemical composition data is provided by refs. ^7,23^. The particle number size distribution data is provided by refs. ^6,24^. All instruments are well calibrated, and datasets are well quality controlled, please refer to the relevant articles for more details. The hourly meteorological data is available from the Indira Gandhi International Airport (https://www.ncdc.noaa.gov/, 8 km away from the supersite), which is well quality controlled according to National Oceanic and Atmospheric Administration, USA^25^.

### Approach for estimating hygroscopicity-induced bias

To estimate the particle hygroscopic growth factor, firstly I use these in-situ observations to estimate the PM_1_ associated Aerosol Liquid Water Content, following the approach detailed in ref. ^16^ Briefly in three steps, i) thermodynamic model ISORROPIA (version 2.1)^26^ is adopted to estimate the inorganic-associated ALWC; ii) the κ-Köhler theory^27,28^ for estimation of organic-associated ALWC, with assumption of organic κ = 0.1 (an index of hygroscopicity) following the previous study in New Delhi^16,17,19^; iii) hydrophobic black carbon with no associated ALWC.

I estimate the sampling underestimation bias via the bulk hygroscopic growth factor of PM_1_ (the ratio between wet and dry diameters) and its particle number size distribution^29^. Assuming that fine particles are homogeneously internally mixed, therefore hygroscopicity is consistent for fine particles with different sizes. This bulk growth factor should well represent the conditions of particles within the range of 100 nm to 1000 nm, because they contribute most to PM_1_ concentrations, and because they have a weak Kelvin effect resulting in a small difference in growth factor (difference < 25% for 80% occasions in Delhi, see 100 nm .vs. 1000 nm in Fig. 3). The collected particles of wet diameter < 1000 nm with corresponding cut-off diameter when it is dry is calculated using the growth factor. Following the method of ref. ^15^, the percentage of mass loss due to sampling-size cut-off shift is estimated using the in-situ observation of fine particle number size distributions^6^.

## Supplementary information

Supplementary information

## Data Availability

The observations of aerosol chemical composition and size distribution in Delhi are available from refs. ^23,24^ under the CC0 licence. The meteorology observations are available from National Oceanic and Atmospheric Administration, USA (https://www.ncdc.noaa.gov/). The PBL conditions are available from ECMWF ERA-interim model (https://www.ecmwf.int/).

## Code availability

Code for calculating PM cutoff shift is available from ref. ^29^.
