# Supplementary material for: Air pollution in New Delhi is more severe than observed due to hygroscopicity-induced bias in aerosol sampling
Source: NPJ Clean Air. 2025 Mar 12;1(1):1. doi: 10.1038/s44407-024-00001-6 (PMC11917838; doi:10.1038/s44407-024-00001-6)
Supplement: Supplementary file 1 — Supplementary information [file 44407_2024_1_MOESM1_ESM.pdf]

Supplementary Materials for

Air pollution in New Delhi is more severe than observed due to hygroscopicity-induced bias in aerosol sampling

Ying Chen[1*]

[1]School of Geography, Earth and Environmental Sciences, University of Birmingham, Edgbaston, Birmingham, B15 2TT, UK

**Correspondence to: Ying Chen (y.chen.21@bham.ac.uk)*

This PDF file includes:

Supplementary Figures 1-2

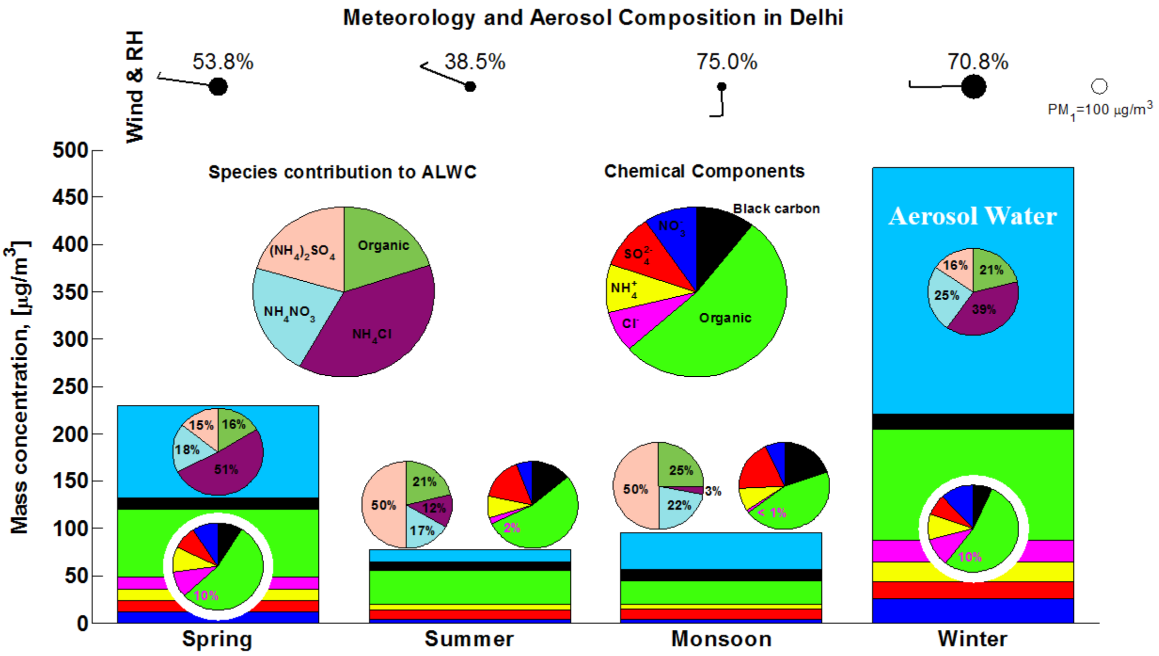

Supplementary Figure 1. Chemical composition of PM_1 in Delhi by season. The average PM_1 mass concentration (size of dot), RH, wind speed and dominant wind direction are given in the top panel. The relative contributions of each chemical component and the aerosol liquid water content (ALWC) associated with it are given in the pie charts. The large pie charts at the top show the average over the whole year. The pale blue colour indicates total ALWC mass concentration. *This figure is sourced from Chen et al. (2022), re-produced under CC-BY 4.0 License.*

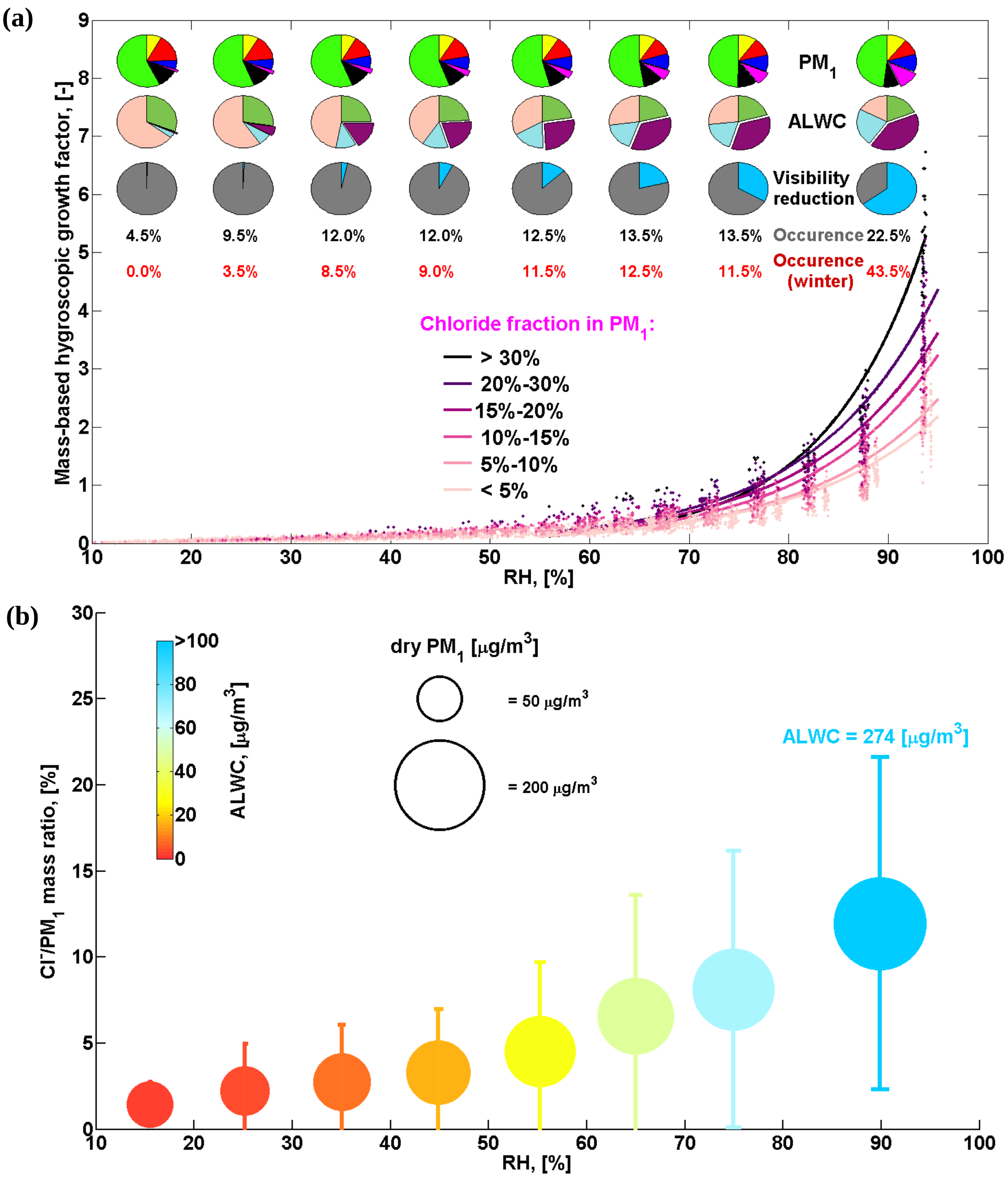

Supplementary Figure 2. Relationships between ALWC, PM_1, chloride fraction and RH. (a) Mass-based hygroscopic growth factor of dry PM_1 (y-axis) as a function of RH for different chloride fractions (indicated by colour). The pie charts show the chemical composition of dry PM_1 (top), the relative contribution of each component to ALWC (middle), and the relative contributions of dry PM_1 (grey) and aerosol water (pale blue) to visibility impairment (bottom). The pie slices for chloride in PM_1 and the contribution of ammonium chloride to ALWC are detached. The colours on the pie charts are the same as in the Supplementary Fig. 1. The frequency of occurrence of each RH regime is marked in black for the whole year and in red for the winter season. (b) Chloride mass fraction in dry PM_1 as a function of RH. ALWC is indicated by colour, and PM_1 dry mass concentration is indicated by the size of circle. The error bars show one standard deviation. *This figure is sourced from Chen et al. (2022), re-produced under CC-BY 4.0 License.*
